# Enhanced Cutaneous Wound Healing *In Vivo* by Standardized Crude Extract of *Poincianella pluviosa*

**DOI:** 10.1371/journal.pone.0149223

**Published:** 2016-03-03

**Authors:** Fernanda Giacomini Bueno, Eduarda Antunes Moreira, Gutierrez Rodrigues de Morais, Isabela Almeida Pacheco, Mauro Luciano Baesso, Eneri Vieira de Souza Leite-Mello, João Carlos Palazzo de Mello

**Affiliations:** 1 Departamento de Farmácia, Palafito, Programa de Pós Graduação em Ciências Farmacêuticas, Universidade Estadual de Maringá, Maringá, Paraná, Brazil; 2 Departamento de Física, Grupo de Fenômenos Fototérmicos, Universidade Estadual de Maringá, Maringá, Paraná, Brazil; 3 Departamento de Ciências Morfológicas, Universidade Estadual de Maringá, Maringá, Paraná, Brazil; Illinois Institute of Technology, UNITED STATES

## Abstract

Wound healing is a complex process that involves several biological events, and a delay in this process may cause economic and social problems for the patient. The search continues for new alternative treatments to aid healing, including the use of herbal medicines. Members of the genus *Caesalpinia* are used in traditional medicine to treat wounds. The related species *Poincianella pluviosa* (DC.) L.P. Queiroz increases the cell viability of keratinocytes and fibroblasts and stimulates the proliferation of keratinocytes *in vitro*. The crude extract (CE) from bark of *P*. *pluviosa* was evaluated in the wound-healing process *in vivo*, to validate the traditional use and the *in vitro* activity. Standardized CE was incorporated into a gel and applied on cutaneous wounds (TCEG) and compared with the formulation without CE (Control) for 4, 7, 10, or 14 days of treatment. The effects of the CE on wound re-epithelialization; cell proliferation; permeation, using photoacoustic spectroscopy (PAS); and proteins, including vascular endothelial growth factor (VEGF), superoxide dismutase 2 (SOD-2) and cyclooxygenase 2 (COX-2) were evaluated. The TCEG stimulated the migration of keratinocytes at day 4 and proliferation on the following days, with a high concentration of cells in metaphase at 7 days. Type I collagen formed more rapidly in the TCEG. PAS showed that the CE had permeated through the skin. TCEG stimulated VEGF at day 4 and SOD-2 and COX-2 at day 7. The results suggest that the CE promoted the regulation of proteins and helped to accelerate the processes involved in healing, promoting early angiogenesis. This led to an increase in the re-epithelialized surface, with significant mitotic activity. Maturation of collagen fibers was also enhanced, which may affect the resistance of the extracellular matrix. PAS indicated a correlation between the rate of diffusion and biological events during the healing process. The CE from *P*. *pluviosa* appears promising as an aid in healing.

## Introduction

The skin forms a barrier that protects the body against intentional or accidental damage such as burns, cuts, abrasions or cutaneous ulcers, which can compromise its function [1,2]. These types of damage are repaired in the wound-healing process, which is very complex and involves several biological events, including vascular and cellular changes, epithelial proliferation, collagen synthesis and deposition, fibroblast proliferation, and wound contraction. However, the time required to complete these stages can change when wound healing is impaired or fails. Nearly 6 million people worldwide are estimated to suffer from unhealed wounds [3].

The cells produce pro-inflammatory cytokines and reactive oxygen species (ROS) such as the superoxide anion and hydrogen peroxide [4,5]. ROS are essential to protect the tissue against microorganisms [5] and stimulate immune cells to release high levels of vascular endothelial growth factor (VEGF) [6]. VEGF induces migration and proliferation of endothelial cells [7]. A “respiratory burst” is caused by an excessive increase in ROS release [8]. Extensive tissue damage including inhibition of cell migration and proliferation can occur if ROS are not detoxified [9]. Superoxide dismutase (SOD), catalase, and some peroxidases are scavengers of these reactive species. Hydrogen peroxide (H_2_O_2_) can be produced by the action of SOD on the superoxide anion. In the endothelial cells, H_2_O_2_ can stimulate the expression of cyclooxygenase 2 (COX-2) and metalloproteinases [10].

A delay in wound repair causes economic and social problems for the patient, raising concerns regarding the reduction in quality of life, mental and physical health, and complications that can cause morbidity and mortality [11]. Due to the high cost of treatment associated with poor wound healing, the search for new drugs to accelerate the healing process has become a priority. For this process to be effective, the wound must close rapidly. A normal healing process should produce a resistant and esthetically satisfying scar [12]. In modern medicine, herbal compounds are assuming an important role in tissue repair. Some reports have described the effects of herbal drugs on wound healing [13,14]. In Brazil, several traditional medicinal plants have been used and studied for their acceleration of wound healing [14,15], although many species remain to be evaluated.

A member of the family Fabaceae, *Poincianella pluviosa* (DC.) L.P. Queiroz is popularly known as "sibipiruna" or "false Brazilwood", and is also reported under its synonyms *Caesalpinia peltophoroides* (Benth.), *Caesalpinia pluviosa* DC., and *Caesalpinia pluviosa* var. *peltophoroides* (Benth.) G.P. Lewis [3,16]. The bark has been investigated as an antimalarial [17] and for its healing activity. Bueno et al. showed that a crude extract of *P*. *pluviosa* increased the *in vitro* cell viability of keratinocytes (HaCaT), and fibroblasts (pNHDF), stimulated the proliferation of keratinocytes and demonstrated the presence of hydrolyzable tannins in the active fraction [16]. In traditional Indian medicine, members of the related genus *Caesalpinia* are used to treat wounds and other injuries [18]. *Caesalpinia* contains about 500 species, and their compounds have diverse biological activities [19]. Several species are used and/or have been evaluated for their healing potential [20,21,22,23].

The present study evaluated the *in vivo* healing effect of a crude extract from the bark of *P*. *pluviosa* on the process of wound re-epithelialization, antioxidant effects, angiogenesis, cell proliferation, and permeation.

## Materials and Methods

### Plant material and crude extract (CE) preparation

Bark of *P*. *pluviosa* was collected on the campus of the Universidade Estadual de Maringá (UEM), Maringá, Paraná, Brazil (23°24'10''S; 51°56'28''W, 564 m a.s.l.). A voucher specimen was deposited in the UEM Herbarium under number HUEM-12492. The bark samples were dried under forced-air circulation (40°C) and then milled in a Tigre ASN-5 stainless-steel hammer mill. Milled bark of *P*. *pluviosa* (10% w/v) was extracted using 50% ethanol (v/v) by turbo-extraction (Ultra-Turrax UTC 115KT, IKA, USA; 15 min; t <40°C). The crude extract (CE) was concentrated in a rotary evaporator under reduced pressure and then lyophilized.

### Antioxidant capacity and total polyphenol content

Antioxidant capacity was estimated based on the DPPH (2,2-diphenyl-1-picrylhydrazyl) radical-scavenging activity, according to the method described by Amarowicz et al., and the results are presented as IC_50_ (μg/mL) [24]. Vitamin C was used as a reference (W.P., China, 100.0%). The CE was standardized according to Bueno et al. [16,25], and the total polyphenol content (TP) was determined using a modified Folin-Ciocalteu method [25].

### Animal experimentation

#### Gel formulation

Two carbopol gel formulations with hydrophilic characteristics, a base gel without CE (BG) and a CE gel containing 1% CE (CEG), were prepared as described by Silva-Corazza et al. and stored at 4–8°C [26]. The formulations were prepared before the beginning of the experiments, and were used during the entire treatment period.

#### Ethics statement and experimental animals

The study was approved by the Animal Ethics Committee of the Universidade Estadual de Maringá (*Permit number*: *141/2010*). Male Wistar rats (*Rattus norvegicus*) weighing 220 to 240 g were kept in individual cages, on a 12-h light/dark cycle, temperature 22°C, with water and chow (Nuvital) *ad libitum*. The animals (n = 34) were divided into four groups, corresponding to 4, 7, 10, and 14 days of treatment. Each group was evaluated by means of histological tests (n = 5), photoacoustic spectroscopy measurements (n = 3), and the Western Blot test (n = 3; except at day 14).

The animals were anesthetized with 2% Rompum (Bayer, São Paulo, Brazil)/10% Ketamine Agener (Agener União, São Paulo, Brazil) (1:1; 0.1 mL/100 g), positioned for a cervical epilation, and two wounds (1 cm^2^ each) were made side by side, by removing the epidermis and dermis. Beginning on the next day, each cutaneous wound was treated daily with base gel on the right side (Control) and CEG on the left side (TCEG). After 4, 7, 10, or 14 days of treatment, the animals were euthanized with an overdose of anesthetic (120 mg/kg Thiopentax, Cristália, São Paulo, Brazil). The cutaneous wounds were examined visually and then skin samples were removed. In the histological test, 2 h before the animals were euthanized, vincristine sulfate (0.5 mg/kg; Tecnocris 1 mg/mL, Zodiac, São Paulo, Brazil) was administered to block mitosis in the epithelial cells.

#### Histological study

The skin samples taken at days 4 and 7 were cut in half. All samples were Bouin-fixed, paraffin-embedded, and cut in semi-serial 6 μm-thick sections. The slides were stained with hematoxylin-eosin (HE) and Sirius red [27]. The re-epithelialization (length and thickness) and number of metaphases were evaluated under an Olympus BX41 light microscope with a 3.2 Megapixel Olympus Q-Color-3 Imaging System coupled to an image capture system (Q-Capture Pro). Types I and III collagen fibers were quantified by the Picro-Sirius technique under an optical microscope coupled to a polarizer (Attachment Nszh-KPO). All slides were analyzed using Image Pro-Plus (v. 4.5).

#### Analyses of re-epithelialization

At days 4 and 7, the upper re-epithelialized surface was measured on each side of the wound. The thickness was evaluated at days 10 and 14, by measuring the re-epithelialized surface at three different points, starting from the center of the wound. Three sections of each slide were analyzed, using a 10X objective. The number of cells in mitosis was determined above the basal and supra-basal surface layers. Five sections of each slide, with a total length of 10,000 μm, were analyzed under a 40X objective. The results were expressed in number of cells in metaphase/mm [28].

#### Collagen fibers

The natural birefringence of collagen, revealed with Picro-Sirius staining and polarized light, allows the types of collagen to be differentiated by their density [27]. The collagen-stained area was calculated by the density of the fibers. Green-stained fibers represent type III collagen, and red-, orange- or yellow-stained fibers represent type I. Three fields were analyzed on each slide, observed using a 20X objective. The results were expressed as percentage of fibers.

#### Analysis of protein by Western Blot

At days 4, 7, or 10, the wounds were removed, fragmented, and homogenized with Tris buffer (50 mmol/L, pH 6.8) containing the protease inhibitors PMSF (10 mg/mL) and aprotinin (2 mg/mL) [29]. The samples were placed in an ultrasonic bath (3 x 15 s) and centrifuged at 4°C. Total protein (μg/mL) present in the supernatant of each sample was measured by the Bradford method [30], and then diluted in a solution containing 1% SDS, 2% 2-mercaptanol, and 10% glycerol, and placed in boiling water for 5 min. Separation and packaging gels containing 10% and 4% polyacrylamide, respectively, and molecular weight standards from 6.9 to 200 kDa (Mark12 Unstained Standard) were used. GAPDH (glyceraldehyde 3-phosphate dehydrogenase) is a constitutive protein and was used as a loading control. After separation by electrophoresis in SDS-PAGE, the proteins were transferred to a nitrocellulose membrane and blocked with Tris buffer solution containing 0.2% Tween-20 (TBST, pH 7.5), and 10% milk protein for 1 h. The membrane was incubated overnight with rabbit monoclonal VEGF, COX-2, SOD-2, and GAPDH (1:250) antibody (Santa Cruz Biotechnology, Santa Cruz, CA), and washed with TBST. The membrane was revealed using a secondary antibody F(ab') 2 fragment of goat anti-mouse IgG (Santa Cruz Biotechnology) conjugated to peroxidase (1:1000) for 1 h. The blot was incubated in a chemiluminescence solution (Novex Chemiluminescent Substrates, Invitrogen) at room temperature, and was autoradiographed with a ChemiDoc XRS System (Bio-Rad). Protein levels were analyzed by densitometry (ImageJ 1.47) and normalized against the GAPDH response (100%).

#### Photoacoustic spectroscopy (PAS) measurements

PAS measurements were carried out according to Rocha et al. [31]. The photoacoustic optical absorption spectra were measured using the light modulation frequency at 22 Hz and scanned the wavelength between 200 and 800 nm. The gel was applied 30 min before the samples were collected and analyzed on the epidermal and dermal surfaces. The absorptions of the CE, BG, and CEG were measured. Spectra obtained on the dermal and epidermal surfaces were subtracted to better assess the skin permeation.

### Statistical analysis

The software Statistica 8.0 (StatSoft, Inc. 1984–2007) was used for the statistical analyses. Data are expressed as mean±standard deviation (SD) using the Mann-Whitney test, a nonparametric analysis for Western Blot; and the Tukey test, a unilateral analysis of variance (one-way ANOVA) for multiple comparisons. Significant differences were determined using *p*<0.05 as the significance criterion.

## Results

**Fig 1:**
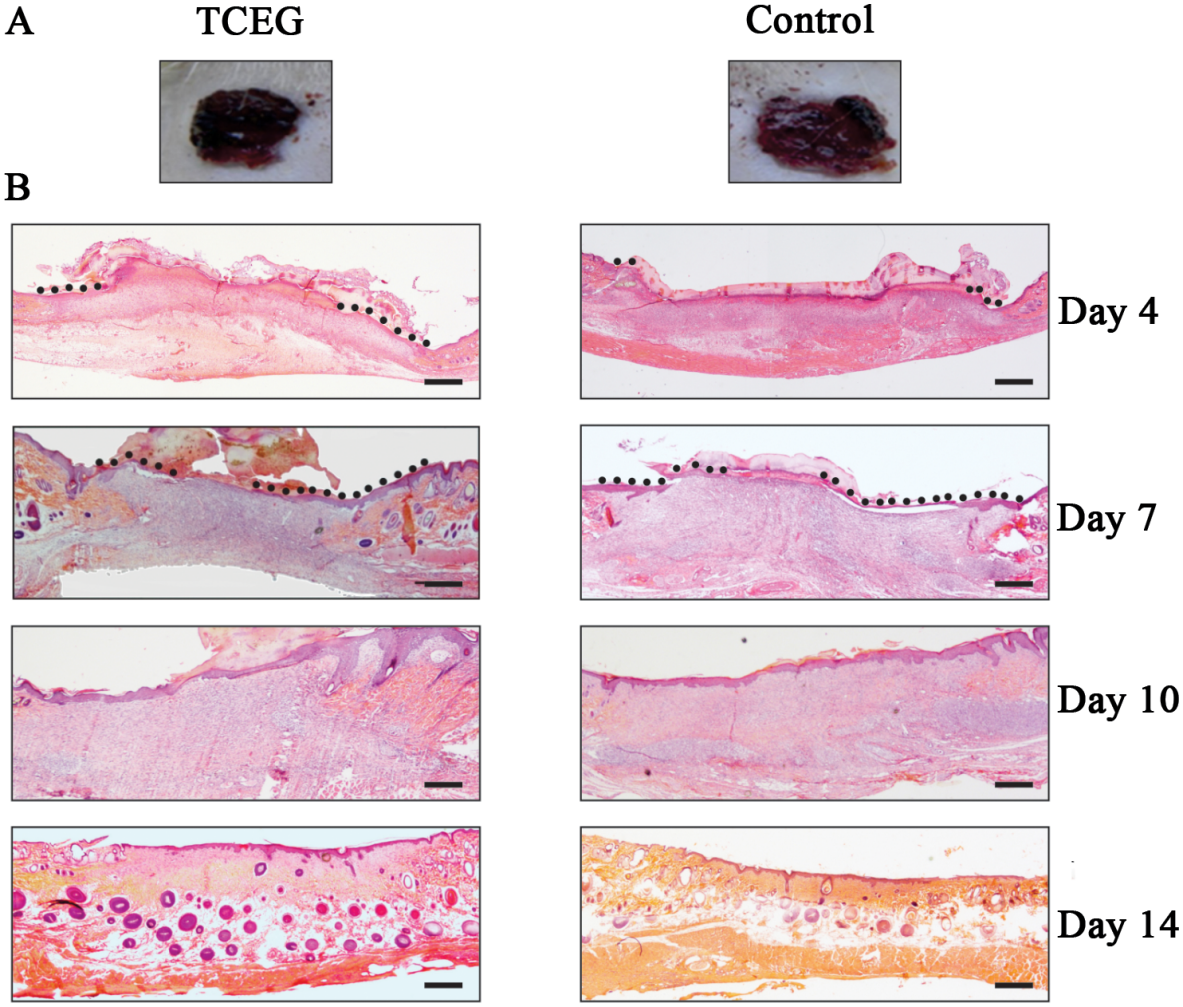
(A) Photographs of excisional wounds on day 2 of treatment: TCEG and Control; (B) Photomicrographs of histological sections stained in hematoxylin-eosin: skin at days 4, 7, 10, and 14 of TCEG and Control. The original magnification was 2x. (●) Representative re-epithelialized surface. (^▬^) 500 μm. TCEG: wound treated with gel containing 1% crude extract; Control: wound treated with base gel.

Antioxidant activity estimated by the DPPH method showed that the inhibitory concentration (IC_50_) was 7.40±0.10 μg/mL for the CE and 4.36±0.08 μg/mL for vitamin C. The total polyphenol content of the CE was 22.7%. The gel formulation was therefore standardized to 22.7 mg% of total polyphenols. The amount of CE in the carbopol gel was optimized from previous studies with *Stryphnodendron adstringens* (barbatimão) [15]. Visual observation of TCEG showed no exudate, inflammation or bleeding on all days of treatment. However, on the second day a rapid browning (darker cherry-red color) and drying crust were observed in the TCEG. In the Control, the crust was less consistent and colored bright red (Fig 1A). These different colors were observed until day 5.

### Histological study

**Fig 2:**
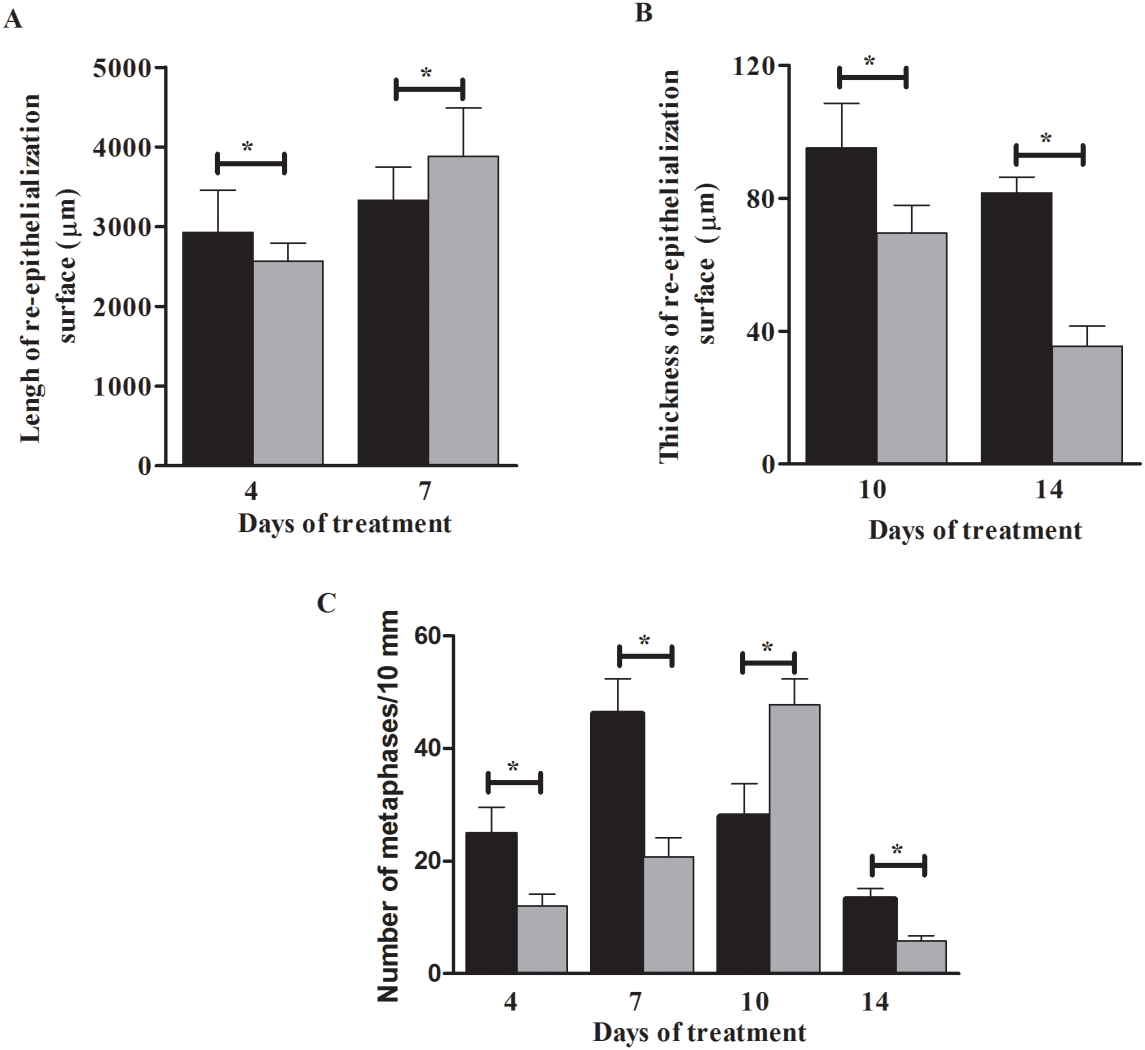
(A) Length at days 4 and 7; (B) thickness at days 10 and 14 of the re-epithelialization surface. (C) Number of cells in metaphase/mm in basal and supra-basal re-epithelialized surface at days 4, 7, 10, and 14. The results are expressed as mean±standard deviation. Values are means of 3 independent experiments. Statistical data were used to compare the days of treatment between TCEG and Control (*p<0.05). (█) TCEG and (█) Control. TCEG: wound treated with gel containing 1% crude extract; Control: wound treated with base gel.

Fig 1B shows the re-epithelialized surface on the days of treatment. Fig 2A and 2B shows the length (at days 4 and 7) and thickness (at days 10 and 14) of the re-epithelialization surface, respectively. At day 4, the length of the re-epithelialized surface at the wound center was greater in the TCEG. At days 10 and 14, the epidermal layer was thicker than that of the Control. At day 7, the re-epithelialized surface peaked in the Control but was thinner than in the TCEG. Statistical analysis showed significant differences (*p*<0.05) on all days in both treatments. In the evaluation of mitotic activity (Fig 2C), at day 4 the TCEG and Control showed 25.0±0.46 and 12.0±0.22 cells in metaphase/10 mm, respectively. At day 7 this number doubled for the treatments, resulting in 45% more cells in metaphase in the TCEG. At day 10, more cells in metaphase were present in the Control, and at day 14 in the TCEG. The results of the treatments were statistically different (*p*<0.05).

**Fig 3:**
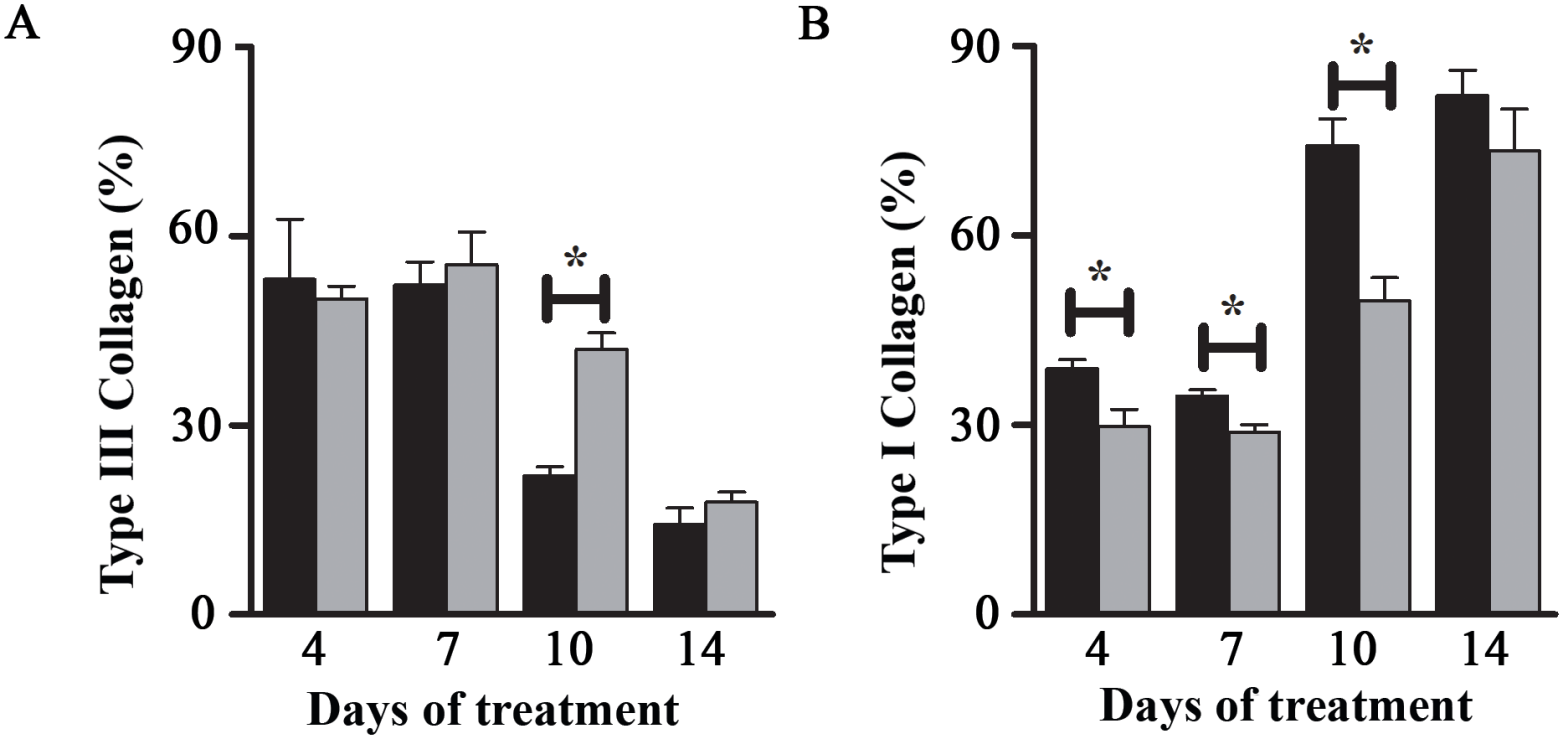
Percentages of (A) type III collagen (immature) and (B) type I collagen (mature) at days 4, 7, 10, and 14. The results are expressed as mean±standard deviation. Values are means of 3 independent experiments. Statistical data were used to compare the days of treatment between TCEG and Control (*p<0.05). (█) TCEG and (█) Control. TCEG: wound treated with gel containing 1% crude extract; Control: wound treated with base gel.

The percentage of type III (immature) collagen was higher at days 4 and 7 for the Control (50.02±1.99 and 56.32±5.61, respectively) as well as for the TCEG (53.14±9.56 and 52.23±3.69, respectively). The percentage decreased on the succeeding days, due to replacement by type I collagen (mature). However, at day 10 there was a significant difference (*p*<0.05) between treatments, with 67% more type I collagen in the TCEG. At day 14 there was re-establishment of the type I collagen in the Control. Fig 3A and 3B shows the collagen on all days of treatment. At days 4, 7, and 10, type I collagen fibers were present in higher percentages (*p*<0.05) in the TCEG compared to the Control (S1 Fig).

### Analysis of protein by Western blot

**Fig 4:**
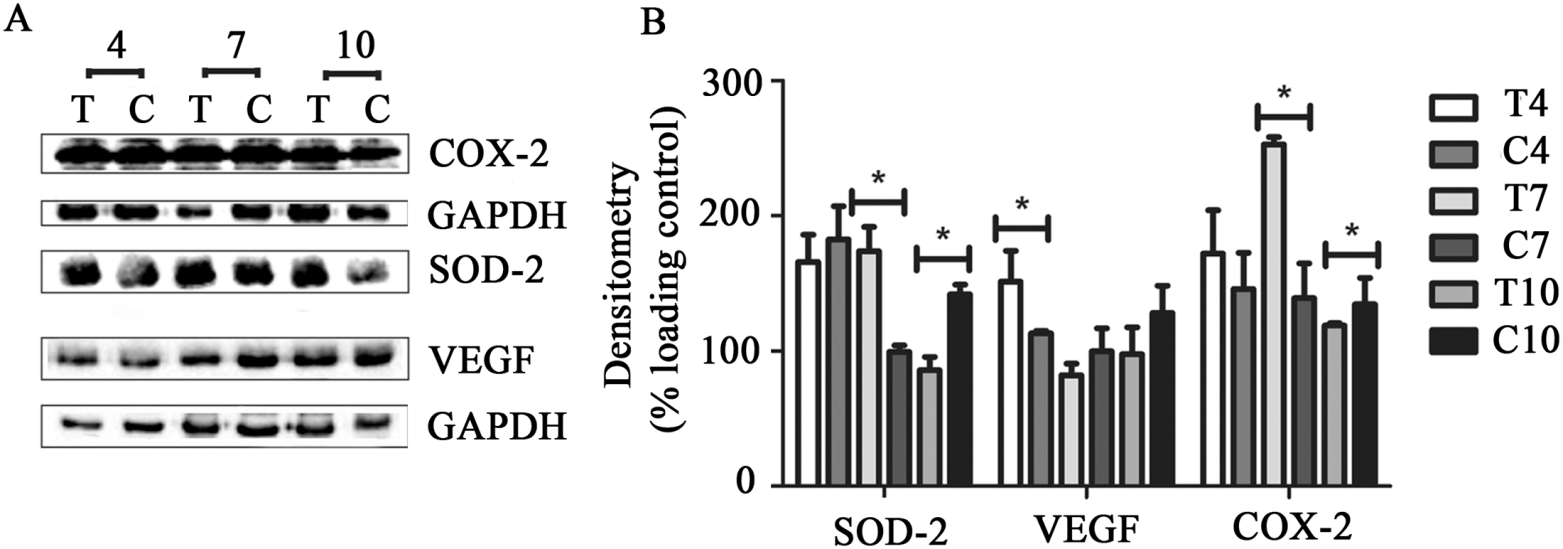
(A) Western blot analyses of COX-2, SOD-2, VEGF, and GAPDH protein at days 4, 7, and 10 of TCEG (T) and Control (C). (B) Measurement obtained from the ratio between optical densities of protein bands from treatments and loading control (GAPDH). GAPDH was set to 100%. The results are expressed as mean±standard deviation. Values are means of 3 independent experiments. Statistical data were used to compare the days of treatment between TCEG and Control (*p<0.05). TCEG: wound treated with gel containing 1% crude extract; Control: wound treated with base gel.

The levels of SOD-2, VEGF, and COX-2 protein was detected at 24, 45, and 70 kDa (Fig 4A), respectively. At day 4, no changes in the protein levels of SOD-2 and COX-2 were observed. In TCEG, VEGF showed a significant (p<0.05) increase at 4 day and SOD-2 and COX-2 showed significant (*p*<0.05) up-regulation at day 7. The proteins returned to basal levels after the maximum peak (Fig 4B).

### Photoacoustic spectroscopy (PAS) measurements

**Fig 5:**
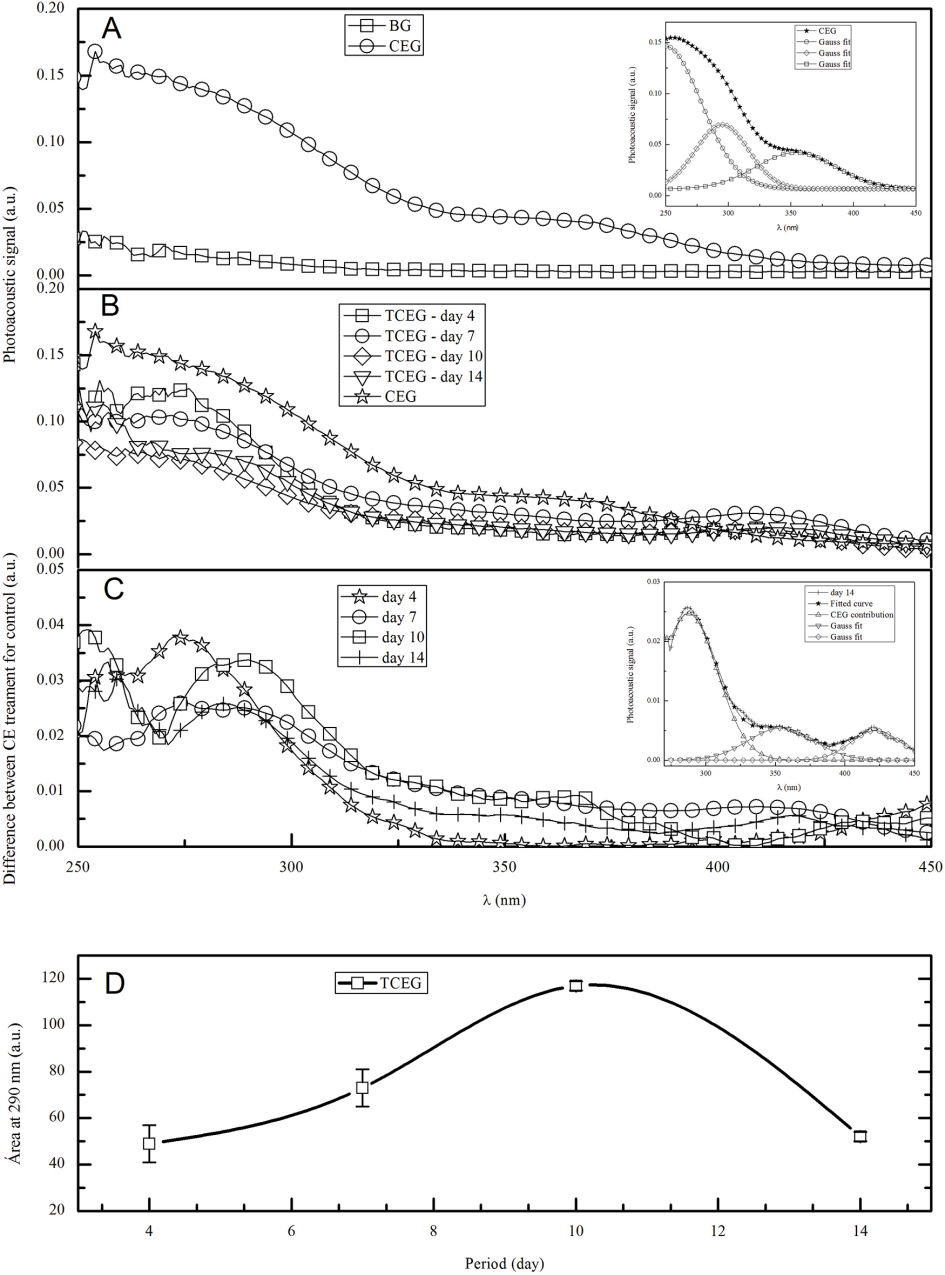
(A) photoacoustic spectra of BG, CEG, and in the inset the Gauss fit of absorption bands of CE; (B) photoacoustic spectra of TCEG over 14 days of treatment; (C) difference between TCEG and control dermal spectra, and in the inset the Gauss fit of absorption bands of CEG; (D) the area of permeation behavior of the absorption band at 290 nm. The results are expressed as mean±standard deviation. Values are means of 3 independent experiments. BG: base gel; CE: crude extract; CEG: gel containing 1% CE; Control: wound treated with BG; TCEG: wound treated with CEG.

The results obtained from photoacoustic spectroscopy showed the spectra of BG and CEG (Fig 5A), where BG has no absorption in the spectral range of 250–450 nm. In Fig 5A-inset, from the determination of Gaussian fit, absorption bands of *P*. *pluviosa* are shown considering the main band at 290 nm. Fig 5B shows the spectra of the dermis treated with CEG, where all spectra have absorption between 250 and 450 nm. Fig 5C shows the subtraction of TCEG dermal spectra from the spectra for the Control dermis, and the presence of bands around 290 nm compared to the band of the CEG. Fig 5C-inset shows the Gaussian fit adjustment of the CEG contribution to the subtraction spectra. The area of this band at 290 nm provides an estimate of the permeation during the healing process (Fig 5D).

## Discussion

Seconds after an injury, hemostasis is triggered with hemorrhage into the wound. Instantly, a blood clot is formed, serving as a physical barrier and producing chemotactic signals [11,32]. The fibrin clot acts as a temporary matrix for cell migration in the next wound-repair stages [1,11]. In our experiment, during the first five days of TCEG, the clot was darker and more consistent than in the Control. This feature can be explained by the presence of polyphenol compounds (22.7%) in CE. Tannins, belonging to the polyphenol group, are able to precipitate with proteins and form a dark crust that covers the wound. They have astringent, antioxidant, and antimicrobial properties [33].

The proliferative phase begins with re-epithelialization involving the extracellular matrix and collagen production [34]. Re-epithelialization closes the wound, reorganizing the cytoskeleton through the migration and proliferation of keratinocytes from the wound edges [35]. As wound closure progresses, epidermal re-epithelialization can be determined by the thickness (days 10 and 14) and length (days 4 and 7) of the re-epithelialization layer. At day 4, the TCEG showed intense migration and proliferation of keratinocytes, and at day 7 the migration was inhibited and proliferation was stimulated. At days 10 and 14. keratinocyte proliferation and differentiation were visible. Cellular proliferation is essential to form a dense hyperproliferative epithelium and restore skin integrity [35]. In the Control, the keratinocyte migration was observed at days 4 and 7, and keratinocyte proliferation at days 10 and 14. Interruption of mitosis at metaphase by the administration of vincristine sulfate, which binds to tubulin and prevents microtubule formation [36], can demonstrate cell proliferation in the TCEG. Mitosis in the TCEG remained high up to day 7, while in the Control, mitosis peaked after day 10, about 3 days later. The results showed that TCEG accelerated re-epithelialization compared to the Control. Values in the re-epithelialization area (after 4 days) and for epidermal thickness (days 10 and 14) were always higher in the TCEG.

While re-epithelialization is proceeding, the extracellular matrix is being laid down. Granulation tissue is the new stroma and consists of fibroblasts, collagen fibers, and new vessels. Fibroblasts are responsible for producing collagen in the temporary extracellular matrix, which is beginning to be replaced by a resistant elastic tissue [34]. At this stage of healing (days 4 and 7), larger amounts of type III (young) collagen were quickly replaced by type I (mature) collagen fibers in the TCEG, with subsequent formation of stronger and less-vascularized tissue [37]. The fibroblast response is related to the increased proliferation of this cell type in this stage of healing [38]. The dermis at days 10 and 14 showed better-organized type I collagen fibers than in the Control. During the fibroblast attachment and maturation, the process of wound contraction reaches its maximum efficiency and the tensile strength may be increased. The ratio of the re-epithelialized area, number of metaphases, and accelerated progression to mature collagen fibers indicate a higher proliferative potential and resistance in wounds treated with the gel containing the CE of *P*. *pluviosa*.

Each stage of wound healing can be regulated by many bioactive compounds including growth factors, cytokines, and eicosanoids. Prostaglandins (PGs), which are inflammatory mediators, belong to the eicosanoid class. Arachidonic acid is converted by cyclooxygenase 1 and 2 (COX) into PGs [39]. The major PG is PGE_2_, which is formed by COX-2, and is involved in keratinocyte proliferation [40], angiogenesis [41], and mediation of the inflammatory response. [42] observed large amounts of COX-2 in the basal layer of wound epidermis and also expressed in inflammatory cells. In this study, COX-2 was stimulated (*p*<0.05) by TCEG, indicating a possible effect on inflammatory cells and keratinocyte proliferation. However, VEGF was stimulated by TCEG at day 4 and returned to basal levels, indicating that *P*. *pluviosa* promoted angiogenesis. VEGF is the most important proangiogenic factor that increases with initial hypoxia [43,44]. The epicatechin gallate, a phenolic compound, stimulated COX-2 and VEGF, improving wound healing in a rat model [45].

Another process that influences wound healing is the presence of large amounts of reactive oxygen species (ROS), which are formed from oxide-reduction reactions during the processes of energy production, phagocytosis, regulation of cell growth, synthesis of substances, and intercellular signaling [46,47]. Free radicals produce numerous disorders, but can be eliminated by antioxidants that are present in the crude extract, which facilitates the healing process [48]. The CE IC_50_ was similar to that of vitamin C; this activity is probably due to phenolic compounds. Another possibility is the production of SOD-2 (day 7), the most common scavenger of ROS found in the mitochondrial matrix [49].

The gel formulation for topical application was also evaluated for permeation of the CE of *P*. *pluviosa* from the dermis to the bloodstream [50]. The PAS spectra were used to determine the penetration profile of the substances through the skin. The resulting bands were evaluated by Gaussian analysis on each day of treatment. The permeation profile was obtained by subtracting the absorption spectrum obtained for the control dermis from the spectrum for the treated dermis, giving the absorption profile for the CE only. As with sunscreens, the gel used to treat skin wounds should have a minimum permeation to the bloodstream, performing its effects in the skin layers [51]. The PAS technique allowed us to determine that the crude extract was absorbed in the wound, showing that the formulation was appropriate to evaluate the wound-healing treatment. The rates of drug diffusion in wounds are affected by the morphological evolution during the repair process, where the most important event for the re-establishment of skin integrity is re-epithelialization. At day 4, the increase in vascularity favored the CE transport into the systemic circulation, as was observed with a propolis extract after 7 days of treatment [15]. Even with the remodeling of the number of blood vessels and the proliferation and differentiation of keratinocytes, the amount of CEG increased until 10 days of treatment. At 14 days, the wound contraction, epidermal reconstitution, and formation of collagen fibers reduce the penetration rate in the skin to a low level [15].

## Conclusion

Use of the formulation containing the crude extract of *P*. *pluviosa* stimulated the formation of collagen fibers and re-epithelialization, indicating that it promotes the formation of more-organized tissue and accelerates wound healing. The up-regulation of proteins by the cells helped to accelerate the processes involved in healing. Phenolic compounds with antioxidant activity present in the CE had a positive effect, providing greater protection for the injured tissue by inhibiting the oxidant agents produced in excess. Photoacoustic spectroscopy allowed us to determine the spread of the formulation during the skin healing, showing a correlation between the rate of diffusion and the biological events of the healing process. Therefore, the CE of *P*. *pluviosa*, applied topically, proved to be a potent promoter of wound repair.

## Supporting Information

S1 Fig Photomicrographs of histological sections stained with Sirius Red: type I collagen (yellow/orange/red) and type III collagen (green) at days 10 and 14, for the TCEG and Control. The original magnification was 2x. (^▬^) 500 μm. TCEG: wound treated with gel containing 1% crude extract; Control: wound treated with base gel. (TIF)

## References

[1] Singer AJ, Clark RA. Cutaneous wound healing. N Engl J Med. 1999;341: 738–746. 10471461 10.1056/NEJM199909023411006

[2] Walter MN, Wright KT, Fuller HR, MacNeil S, Johnson WE. Mesenchymal stem cell-conditioned medium accelerates skin wound healing: an *in vitro* study of fibroblast and keratinocyte scratch assays. Exp Cell Res. 2010;316: 1271–1281. 10.1016/j.yexcr.2010.02.026 20206158

[3] Kumar B, Vijayakumar M, Govindarajan R, Pushpangadan P. Ethnopharmacological approaches to wound healing-exploring medicinal plants of India. J ethnopharmacol. 2007;114: 103–113. 17884316 10.1016/j.jep.2007.08.010

[4] Schafer M, Werner S. Oxidative stress in normal and impaired wound repair. Pharmacol Res. 2008;58: 165–171. 10.1016/j.phrs.2008.06.004 18617006

[5] Werner S, Grose R. Regulation of wound healing by growth factors and cytokines. Physiol Rev. 2003;83: 835–870. 12843410 10.1152/physrev.2003.83.3.835

[6] Cho M, Hunt TK, Hussain MZ. Hydrogen peroxide stimulates macrophage vascular endothelial growth factor release. Am J Physiology. 2001;280: H2357–2363. 10.1152/ajpheart.2001.280.5.H2357 11299242

[7] Bao P, Kodra A, Tomic-Canic M, Golinko MS, Ehrlich HP, Brem H. The role of vascular endothelial growth factor in wound healing. J Surg Res. 2009;153: 347–358. 10.1016/j.jss.2008.04.023 19027922 PMC2728016

[8] Keller U, Kümin A, Braun S, Werner S. Reactive oxygen species and their detoxification in healing skin wounds. J Investig Dermatol Symp Proc. 2006;11: 106–111. 17069017 10.1038/sj.jidsymp.5650001

[9] Kanta J. The role of hydrogen peroxide and other reactive oxygen species in wound healing. Acta Medica. 2011;54: 97–101. 22250477 10.14712/18059694.2016.28

[10] Schremi S, Landthaler M, Shäferling M, Babilas P. A new star on the H2O2rizon of wound healing? Exp Dermatol. 2010;20: 229–231. 10.1111/j.1600-0625.2010.01195.x 21323744

[11] Velnar T, Bailey T, Smrkolj V. The wound healing process: an overview of the cellular and molecular mechanisms. J Int Med Res. 2009;37: 1528–1542. 19930861 10.1177/147323000903700531

[12] Tam JC, Lau KM, Liu CL, To MH, Kwok HF, Lai KK et al The *in vivo* and *in vitro* diabetic wound healing effects of a 2-herb formula and its mechanisms of action. J Ethnopharmacol. 201;134: 831–838. 10.1016/j.jep.2011.01.032 21291991

[13] Nayak BS, Sandiford S, Maxwell A. Evaluation of the wound-healing activity of ethanolic extract of *Morinda citrifolia* L. leaf. Evid Based Complement Alternat Med. 2009;6: 351–356. 10.1093/ecam/nem127 18955257 PMC2722214

[14] Schmidt C, Fronza M, Goettert M, Geller F, Luik S, Flores EM et al Biological studies on Brazilian plants used in wound healing. J Ethnopharmacol. 2009;122: 523–532. 10.1016/j.jep.2009.01.022 19429323

[15] Hernandes L, Pereira LMS, Palazzo F, Mello JCP. Wound-healing evaluation of ointment from Stryphnodendron adstringens (barbatimão) in rat skin. Braz J Pharm Sci. 2010;46: 431–436.

[16] Bueno FG, Panizzon GP, Mello EV, Lechtenberg M, Petereit F, Mello JCP et al Hydrolyzable tannins from hydroalcoholic extract from *Poincianella pluviosa* stem bark and its wound-healing properties: phytochemical investigations and influence on *in vitro* cell physiology of human keratinocytes and dermal fibroblasts. Fitoterapia. 2014;99: 252–260. 10.1016/j.fitote.2014.10.007 25454458

[17] Kayano AC, Lopes SC, Bueno FG, Cabral EC, Souza-Neiras WC, Yamauchi LM et al In vitro and in vivo assessment of the anti-malarial activity of *Caesalpinia pluviosa*. Malar J. 2011;10: 112 10.1186/1475-2875-10-112 21535894 PMC3112450

[18] Khare CP. Indian herbal remedies: rational western therapy, ayurvedic and other traditional usage, botany; New York: Heidelberg S-VB, editor; 2004.

[19] Zanin JL, de Carvalho BA, Martineli PS, dos Santos MH, Lago JH, Sartorelli P et al The genus Caesalpinia L. (Caesalpiniaceae): phytochemical and pharmacological characteristics. Molecules. 2012;17: 7887–7902. 10.3390/molecules17077887 22751225 PMC6269049

[20] Chopda MZ, Mahajan RT. Wound healing plants of Jalgaon district of Maharashta State, India. Ethnobot Leaflets. 2009;13: 1–32.

[21] An K, Nayeem N. Formulation and evaluation of the methanolic extract of Caesalpinia pulcherrima leaves for its wound healing activity. Asian J Pharmaceut Res Health Care. 2012;4: 90–94.

[22] Patil KS. Wound healing acticity of the seed kernels of Caesalpinia crista Linn. J Nat Remedies. 2005;5: 26–30.

[23] Tewtrakul S, Tungcharoen P, Sudsai T, Karalai C, Ponglimanont C, Yodsaoue O. Antiinflammatory and wound healing effects of Caesalpinia sappan L. Phytother Res. 2015;29: 850–856. 10.1002/ptr.5321 25760294

[24] Amarowicz R, Pegg RB, Rahimi-Moghaddam P, Barl B, Weil J.A. Free-radical scavenging capacity and antioxidant activity of selected plant species from the Canadian prairies. Food Chem. 2004;84: 551–562.

[25] Bueno FG, Machareth MAD, Panizzon GP, Lopes G.C., Mello EVSL, Mello JCP. Development of a UV/Vis spectrophotometric method for analysis of total polyphenols from *Caesalpinia peltophoroides* Benth. Quim Nova. 2012;32: 822–826.

[26] Silva-Corazza PER, Lopes GC, Diciaula MC, Lima MMS, Mello JCP. Pharmaceutical topical gel: development and validation of a UV spectrophotometric method for determination of polyphenols. Lat Am J Pharm. 2010;29: 830–834.

[27] Dayan D, Hiss Y, Hirshberg A, Bubis JJ, Wolman M. Are the polarization colors of picrosirius red-stained collagen determined only by the diameter of the fibers? Histochemistry. 1989;93: 27–29. 2482274 10.1007/BF00266843

[28] Sehn E, Hernandes L, Franco SL, Goncalves CC, Baesso ML. Dynamics of reepithelialisation and penetration rate of a bee propolis formulation during cutaneous wounds healing. Anal Chim Acta. 2009;635: 115–120. 10.1016/j.aca.2009.01.019 19200487

[29] Gupta A, Upadhyay NK, Sawhney RC, Kumar R. A poly-herbal formulation accelerates normal and impaired diabetic wound healing. Wound Repair Regen. 2008;16: 784–790. 10.1111/j.1524-475X.2008.00431.x 19128249

[30] Bradford MM. A rapid and sensitive method for the quantitation of microgram quantities of protein utilizing the principle of protein-dye binding. Anal Biochem. 1976;72: 248–254. 942051 10.1016/0003-2697(76)90527-3

[31] Rocha JC, Pedrochi F, Hernandes L, de Mello JC, Baesso ML. *Ex vivo* evaluation of the percutaneous penetration of proanthocyanidin extracts from *Guazuma ulmifolia* using photoacoustic spectroscopy. Anal Chim Acta. 2007;587: 132–136. 17386764 10.1016/j.aca.2007.01.002

[32] Gantwerker EA, Hom DB. Skin: histology and physiology of wound healing. Facial Plast Surg Clin North Am. 2011;19: 441–453. 10.1016/j.fsc.2011.06.009 21856533

[33] Agyare C, Bempah SB, Boakye YD, Ayande PG, Adarkwa-Yiadom M, Mensah KB. Evaluation of antimicrobial and wound healing potential of *Justicia flava* and *Lannea welwitschii*. Evid Based Complement Alternat Med. 2013; 632927 10.1155/2013/632927 24159350 PMC3789403

[34] Gentleman E, Lay AN, Dickerson DA, Nauman EA, Livesay GA, Dee KC. Mechanical characterization of collagen fibers and scaffolds for tissue engineering. Biomaterials. 2003;24: 3805–3813. 12818553 10.1016/s0142-9612(03)00206-0

[35] Santoro MM, Gaudino G. Cellular and molecular facets of keratinocyte reepithelization during wound healing. Experim Cell Res. 2005;304: 274–286. 10.1016/j.yexcr.2004.10.033 15707592

[36] Mujagic H, Chen SS, Geist R, Occhipinti SJ, Conger BM, Smith CA et al Effects of vincristine on cell survival, cell cycle progression, and mitotic accumulation in asynchronously growing Sarcoma 180 cells. Cancer Res. 1983;43: 3591–3597. 6861131

[37] Gonçalves RV, Mezencio JM, Benevides GP, Matta SL, Neves CA, Sarandy MM et al Effect of gallium-arsenide laser, gallium-aluminum-arsenide laser and healing ointment on cutaneous wound healing in Wistar rats. Braz J Med Biol Res. 2010;43: 350–355. 10.1590/S0100-879X2010007500022 20445949

[38] Smith AN, Willis E, Chan VT, Muffley LA, Isik FF, Gibran NS et al Mesenchymal stem cells induce dermal fibroblast responses to injury. Experim Cell Res. 2010;316: 48–54. 10.1016/j.yexcr.2009.08.001 PMC2787776 19666021

[39] Wada M, DeLong CJ, Hong YH, Rieke CJ, Song I, Sidhu RS et al Enzymes and receptors of prostaglandin pathways with arachidonic acid-derived versus eicosapentaenoic acid-derived substrates and products. J Biol Chem. 2007;282: 22254–22266. 17519235 10.1074/jbc.M703169200

[40] Pentland AP, Needleman P. Modulation of keratinocyte proliferation in vitro by endogenous prostaglandin synthesis. J Clin Invest. 1986;77: 246–251. 3080474 10.1172/JCI112283 PMC423333

[41] Form DM, Auerbach R. PGE2 and angiogenesis. Proc Soc Exp Biol Med. 1983;172: 214–218. 6572402 10.3181/00379727-172-41548

[42] Futagami A, Ishizaki M, Fukuda Y, Kawana S, Yamanaka N. Wound healing involves induction of cyclooxygenase-2 expression in rat skin. Lab Invest. 2002;82: 1503–1513. 12429810 10.1097/01.lab.0000035024.75914.39

[43] Sano H, Ichioka S, Sekiya N. Influence of oxygen on wound healing dynamics: assessment in a novel wound mouse model under a variable oxygen environment. PloS one. 2012;7: e50212 10.1371/journal.pone.0050212 23209678 PMC3509142

[44] Tandara AA, Mustoe TA. Oxygen in wound healing-more than a nutrient. World J Surg. 2004;28: 294–300. 14961188 10.1007/s00268-003-7400-2

[45] Kapoor M, Howard R, Hall I, Appleton I. Effects of epicatechin gallate on wound healing and scar formation in a full thickness incisional wound healing model in rats. Am J Pathol. 2004;165: 299–307. 15215184 10.1016/S0002-9440(10)63297-X PMC1618547

[46] Ratman D, Ankola DD, Bhardwaj V, Sahara DK, Kumar MNV. Role of antioxidants in prophylaxis and therapy: a pharmaceutical perspective. J Control Release. 2006;113: 189–207. 16790290 10.1016/j.jconrel.2006.04.015

[47] Gülçin I, Huyut Z, Elmastas M, Aboul-Enein HY. Radical scavenging and antioxidant activity of acid. J Chem. 2010;3: 43–53.

[48] Tawaha K, Alali FQ, Gharaibeh M, Mohammad M, El-Elimat T. Antioxidant activity and total phenolic content of selected Jordanian plant species. Food Chem. 2007;104: 1372–1378.

[49] Abe M, Saitoh H, Sato Y, Hamaguchi K, Kiuchi M. Immunohistochemical study of the kidneys after severe muscular injury. Int J Legal Med. 2001;114: 232–236. 11355401 10.1007/s004140000170

[50] Barry BW. Novel mechanisms and devices to enable successful transdermal drug delivery. Eur J Pharm Sci. 2001;14: 101–114. 11500256 10.1016/s0928-0987(01)00167-1

[51] Gupta VK, Zatz JL, Rerek M. Percutaneous absorption of sunscreens through micro-yucatan pig skin in vitro. Pharmaceut Res. 1999;16: 1602–1607. 10.1023/a:1018916907263 10554104

