# Supplementary figures and images for: Enhanced Cutaneous Wound Healing In Vivo by Standardized Crude Extract of Poincianella pluviosa
Source: PLoS One. 2016 Mar 3;11(3):e0149223. doi: 10.1371/journal.pone.0149223 (PMC4777426; doi:10.1371/journal.pone.0149223)

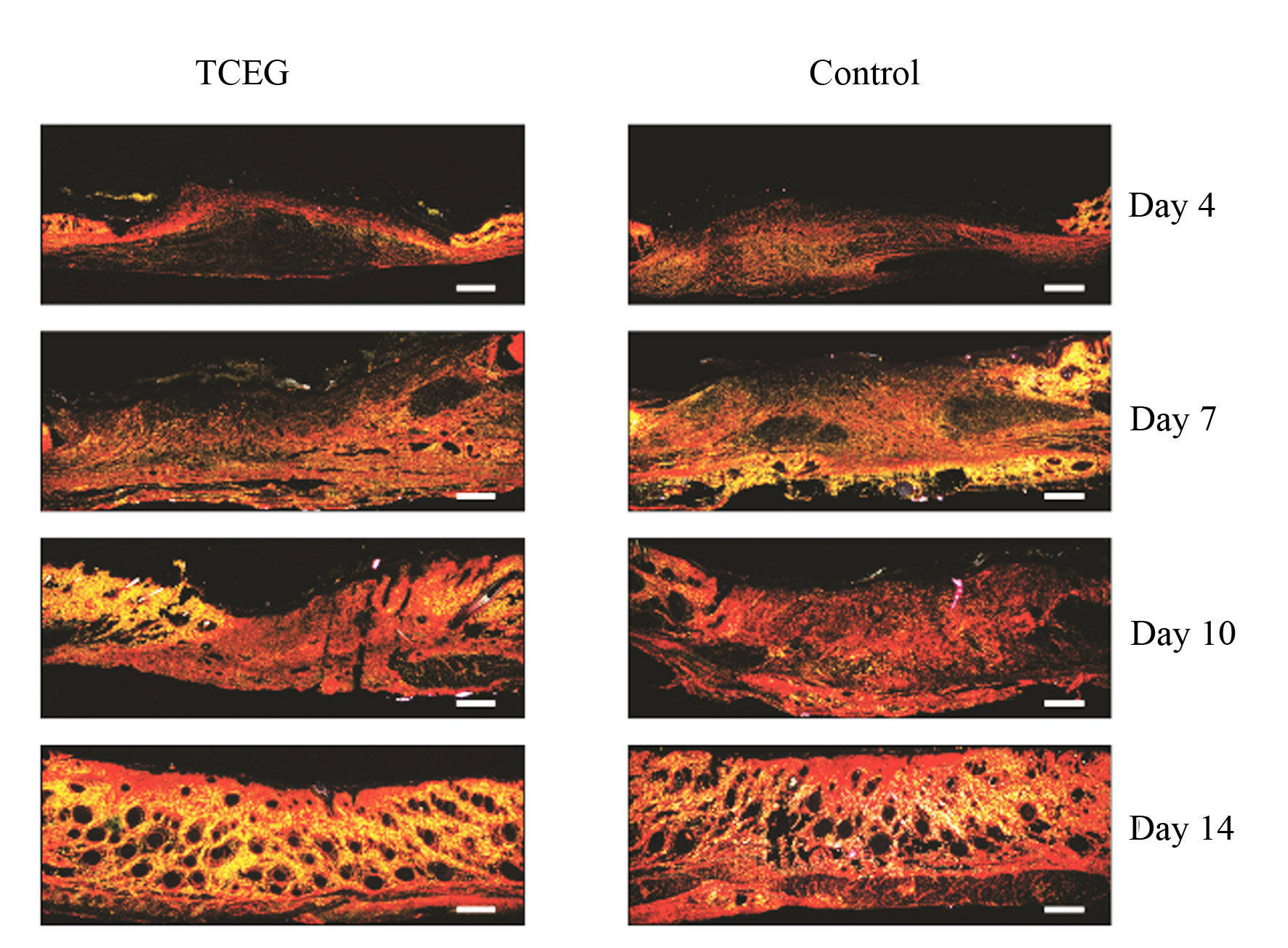

Supplement: S1 Fig — (TIF) [file pone.0149223.s001.tif]
